# Nrf2 activation does not affect adenoma development in a mouse model of colorectal cancer

**DOI:** 10.1038/s42003-021-02552-w

**Published:** 2021-09-15

**Authors:** Elena V. Knatko, Cecilia Castro, Maureen Higgins, Ying Zhang, Tadashi Honda, Colin J. Henderson, C. Roland Wolf, Julian L. Griffin, Albena T. Dinkova-Kostova

**Affiliations:** 1 Jacqui Wood Cancer Centre, Division of Cellular Medicine, School of Medicine, University of Dundee, Dundee, Scotland UK; 2 Department of Biochemistry and the Cambridge Systems Biology Centre, University of Cambridge, Cambridge, UK; 3 Department of Chemistry and Institute of Chemical Biology & Drug Discovery, Stony Brook University, Stony Brook, NY USA; 4 Division of Systems Medicine, School of Medicine, University of Dundee, Dundee, Scotland UK; 5 Section of Biomolecular Medicine, Department of Metabolism, Digestion and Reproduction, Imperial College London, London, UK; 6 Department of Pharmacology and Molecular Sciences and Department of Medicine, Johns Hopkins University School of Medicine, Baltimore, MD USA

**Keywords:** Cancer, Drug discovery

## Abstract

Transcription factor nuclear factor erythroid 2 p45-related factor 2 (Nrf2) and its main negative regulator, Kelch-like ECH associated protein 1 (Keap1), are at the interface between redox and intermediary metabolism. Nrf2 activation is protective in models of human disease and has benefits in clinical trials. Consequently, the Keap1/Nrf2 protein complex is a drug target. However, in cancer Nrf2 plays a dual role, raising concerns that Nrf2 activators may promote growth of early neoplasms. To address this concern, we examined the role of Nrf2 in development of colorectal adenomas by employing genetic, pharmacological, and metabolomic approaches. We found that colorectal adenomas that form in Gstp^−/−^: Apc^Min/+^ mice are characterized by altered one-carbon metabolism and that genetic activation, but not disruption of Nrf2, enhances these metabolic alterations. However, this enhancement is modest compared to the magnitude of metabolic differences between tumor and peri-tumoral tissues, suggesting that the metabolic changes conferred by Nrf2 activation may have little contribution to the early stages of carcinogenesis. Indeed, neither genetic (by Keap1 knockdown) nor pharmacological Nrf2 activation, nor its disruption, affected colorectal adenoma formation in this model. We conclude that pharmacological Nrf2 activation is unlikely to impact the early stages of development of colorectal cancer.

## Abstract (web summary)

Knatko et al. examined the importance of Nrf2 activity in the development of colorectal adenomas by performing genetic and pharmacological analyses. The authors found that Keap1 knockdown and Nrf2 activation impacted the metabolism of the non-tumoral colon, but did not affect colorectal adenoma formation in this model.

## Introduction

To adapt to conditions of oxidative, electrophilic, and inflammatory stress, cells have evolved networks of cytoprotective proteins, the gene expression of which is controlled by transcription factor nuclear factor erythroid 2 p45-related factor 2 (Nrf2) and its main negative regulator the Kelch-like ECH associated protein 1 (Keap1)^1^. Activation of Nrf2 has shown protective effects in numerous animal models of chronic disease^2^ and has beneficial effects in human clinical trials^3^. Nrf2 is considered a drug target, and several small molecule Nrf2 inducers are currently at various stages of drug development^4^. Among them, the naturally occurring isothiocyanate sulforaphane, the semi-synthetic pentacyclic cyanoenone triterpenoids, and their tricyclic derivatives are the most potent Nrf2 inducers known to date^5–8^. The cyanoenones activate Nrf2 at low nanomolar concentrations by modifying C151 in Keap1^9,10^. The tricyclic cyanoenone TBE-31 is highly bioavailable^11^, has a covalent and reversible mode of action, and is suitable for chronic oral administration^12^. Furthermore, TBE-31 has demonstrated protective effects in mouse models of cutaneous squamous cell carcinoma^13^, and non-alcoholic steatohepatitis^14^ and in a rat model of aflatoxin-induced hepatocarcinogenesis^6^.

Although Nrf2 is primarily regulated at the protein stability level, the expression of *NFE2L2*, the gene encoding Nrf2, is another important determinant of cellular Nrf2 levels, which has been shown to affect susceptibility to disease. Thus, a single nucleotide polymorphism (SNP) in the human *NFE2L2* promoter lowers its gene expression and increases the risk for lung cancer^15^. However, persistent activation of Nrf2 is frequently exploited by cancer cells, where it promotes their survival and resistance to chemotherapy and radiation therapy^16,17^, and contributes to metabolic adaptation^18^. Mutations in Keap1 or Nrf2, which abrogate formation of the Keap1/Nrf2 protein complex or prevent Nrf2 ubiquitination, leading to its constitutive activation, occur in several types of human cancer, and are particularly prominent in squamous cell carcinomas of the lung, contributing to tumor growth and resistance to chemo- and radiation therapy^19,20^. Importantly, it was recently shown in the context of non-small cell lung cancer that constitutively active Nrf2 generates enhancers at gene loci that are not normally regulated following transient activation of Nrf2 under physiological conditions^21^. Nrf2 is also upregulated in human colorectal tumors, where high levels of nuclear Nrf2 correlate with poor patient prognosis^22^. However, whether Nrf2 affects the early stages of adenoma development in the colon is unclear.

It is well established that in human colorectal cancers, the adenomatous polyposis coli (*APC*) gene is frequently mutated^23^ or inactivated by promoter hypermethylation^24^, and its germline mutations cause familial adenomatous polyposis (FAP), an autosomal dominant inherited condition in which numerous adenomas form in the epithelium of the large intestine^25^. Similar to humans, Apc^Min/+^mice that have a mutant allele encoding a nonsense mutation at codon 850 of the murine *Apc* gene, are predisposed to intestinal adenoma formation, but unlike in humans, the tumors in mice form predominantly in the small intestine^26^. Interestingly, Apc^Min/+^mice that are also deficient for glutathione transferase Pi (Gstp^−/−^: Apc^Min/+^) have a 6-fold increase in colon adenoma incidence, and a 50-fold increase in colorectal adenoma multiplicity, in comparison with their Gstp wild-type (Gstp^+/+^: Apc^Min/+^) counterparts^27^. Using the Gstp^−/−^: Apc^Min/+^ mouse model, in this study we examined the role of Nrf2 in the development of colorectal adenomas by employing genetic and pharmacological approaches. We then used metabolomics to characterize tumorous and non-tumorous tissue from mice with different levels of Nrf2.

## Results

### The expression of classical Nrf2 targets and pro-inflammatory genes is not affected by deletion of Gstp

**Fig. 1:**
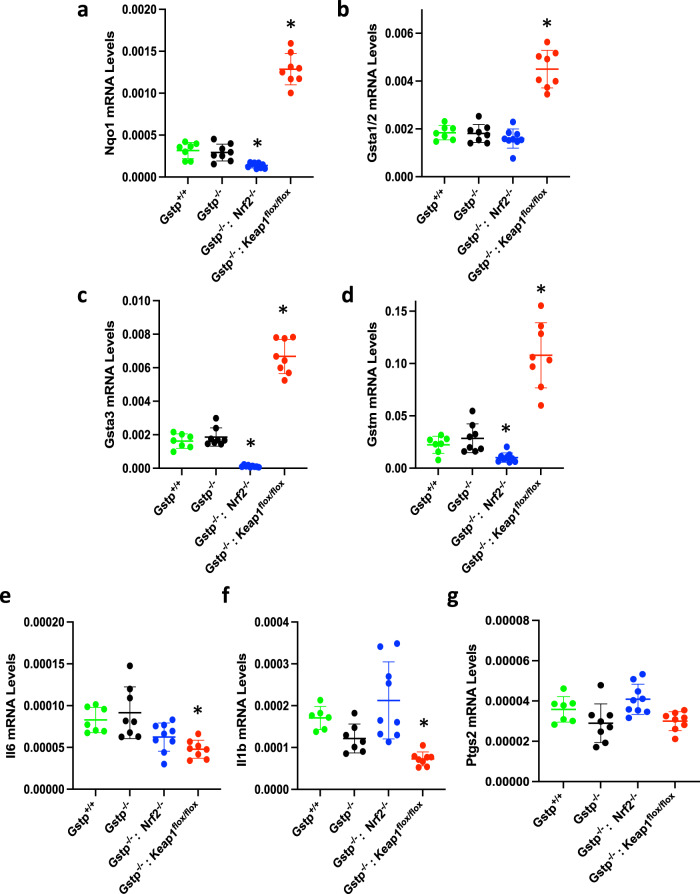
The expression of classical Nrf2 targets and pro-inflammatory genes is not affected by deletion of *Gstp*. mRNA levels for Nqo1 (**a**), Gsta1/2 (**b**), Gsta3 (**c**), Gstm (**d**), Il6 (**e**) Il1β (**f**), and Ptgs2 (**g**) in colon tissue of Gstp^+/+^: Nrf2^+/+^: Keap1^+/+^ (green circles), Gstp^−/−^: Nrf2^+/+^: Keap1^+/+^ (black circles), Gstp^−/−^: Nrf2^−/−^: Keap1^+/+^ (blue circles), and Gstp^−/−^: Nrf2^+/+^: Keap1^flox/flox^ (red circles) female mice (*n* = 8). Results are expressed as relative values to the Gstp^+/+^: Nrf2^+/+^: Keap1^+/+^ genotype. Actb was used as the reference gene. *p < 0.01 (compared to Gstp^+/+^: Nrf2^+/+:^ Keap1^+/+^).

Because Gstp is involved in xenobiotic metabolism^28^ as well as cell signaling, by sequestering c-Jun N-terminal kinase (JNK)^29^, we first asked whether deletion of Gstp affects the Nrf2-mediated transcription using the classical Nrf2-target NAD(P)H:quinone oxidoreductase 1 (Nqo1) as a marker, which is expressed in an Nrf2-dependent manner in the mouse colon^30^. The mRNA levels for Nqo1 in the colon tissues did not differ between Gstp^+/+^ and Gstp^−/−^ animals (Fig. 1a). As expected, these levels were ~60% lower in mice with disrupted Nrf2 (Gstp^−/−^: Nrf2^−/−^) and ~4-fold higher in mice with high Nrf2 levels due to Keap1 downregulation (Gstp^−/−^: Keap1^flox/flox^) **(**Fig. 1a).

The GSTs are a large family of detoxification enzymes, and we next asked whether deletion of Gstp affected the expression of other members of the GST family. The mRNA levels in the colon for Gsta1/2 (Fig. 1b), Gsta3 (Fig. 1c), and Gstm (Fig. 1d) did not differ significantly between Gstp^−/−^: Nrf2^+/+^: Keap1^+/+^ and Gstp^+/+^: Nrf2^+/+^: Keap1^+/+^ mice. As expected based on published work comparing their expression in the intestine of wild-type and Nrf2-knockout mice^31^, the expression levels of Gsta3 and Gstm were lower in Nrf2^−/−^ and higher in Keap1^flox/flox^ mice, in comparison with their Nrf2^+/+^ and Keap1^+/+^ counterparts **(**Fig. 1c, d). By contrast, the mRNA levels for the pro-inflammatory cytokines interleukin-6 (Il6) **(**Fig. 1e) and Il1β **(**Fig. 1f) were lower in Keap1^flox/flox^ mice. The basal levels of expression of prostaglandin-endoperoxide synthase 2 (Ptgs2), more commonly known as cyclooxygenase 2 (Cox2), were not affected by Gstp deletion, Nrf2 disruption or Keap1 knockdown **(**Fig. 1g).

### Genetic activation or disruption of Nrf2 does not affect colorectal adenoma formation in Gstp^−/−^: Apc^Min/+^ mice

**Fig. 2:**
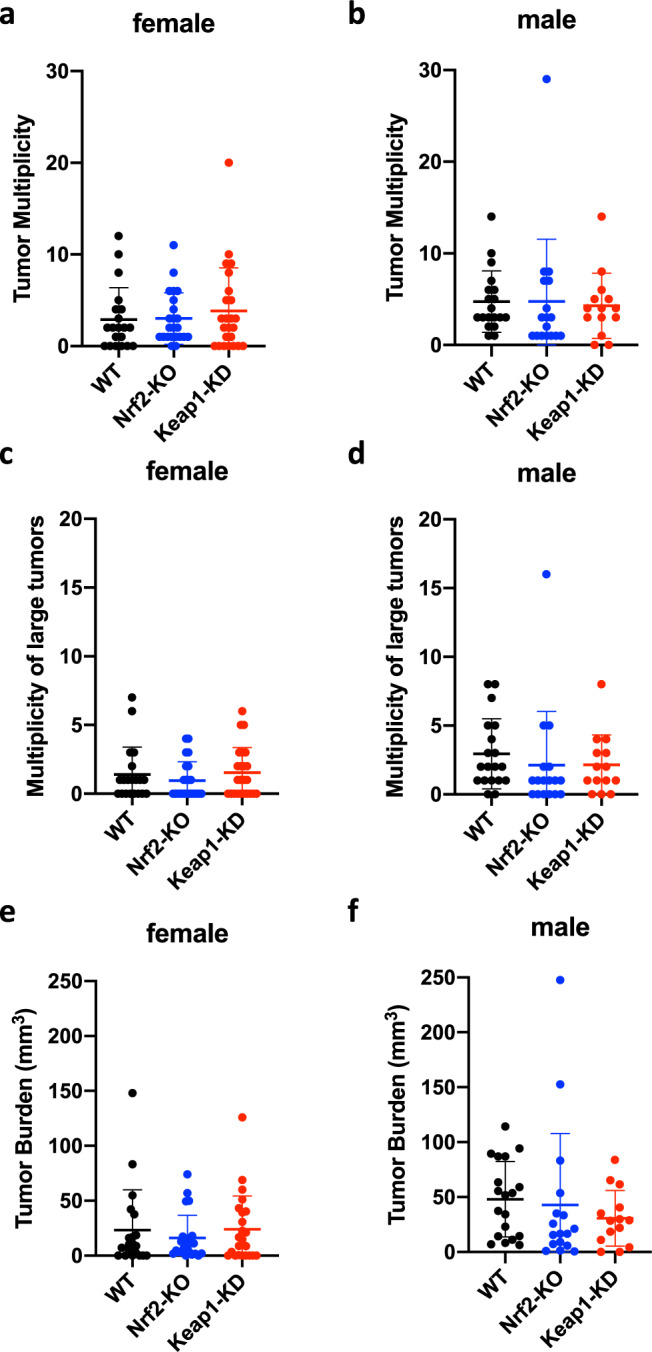
Genetic activation or disruption of Nrf2 does not affect colorectal adenoma formation in Gstp^−/−^: Apc^Min/+^ mice. Tumor multiplicity (**a**–**d**) and burden (**e**, **f**) in colons of 20-week-old Gstp^−/−^: Apc^Min/+^: Nrf2^+/+^: Keap1^+/+^ (WT, black circles), Gstp^−/−^: Apc^Min/+^: Nrf2^−/−^: Keap1^+/+^(Nrf2-KO, blue circles), and Gstp^−/−^: Apc^Min/+^: Nrf2^+/+^: Keap1^flox/flox^ (Keap1-KD, red circles) mice (*n* = 38–40).

To address whether genetic activation or disruption of Nrf2 affects the development of colorectal tumors, we generated Gstp^−/−^: Apc^Min/+^: Nrf2^−/−^: Keap1^+/+^ and Gstp^−/−^: Apc^Min/+^: Nrf2^+/+^: Keap1^flox/flox^ mice by crossing Gstp^−/−^: Apc^Min/+^ mice with Nrf2^−/−^ or Keap1^flox/flox^ mice, respectively, all on the C57BL/6 genetic background. For simplicity, throughout the rest of the manuscript, we refer to these genotypes of mice as follows: Gstp^−/−^: Apc^Min/+^: Nrf2^+/+^: Keap1^+/+^ = wild-type (WT); Gstp^−/−^: Apc^Min/+^: Nrf2^−/−^: Keap1^+/+^ = Nrf2-knockout (Nrf2-KO); and Gstp^−/−^: Apc^Min/+^: Nrf2^+/+^: Keap1^flox/flox^ mice = Keap1-knockdown (Keap1-KD). The number and volume of colon neoplasms (>1 mm height) were assessed at 20 weeks (when due to welfare concerns, many animals had to be euthanized) in order to establish whether genetic activation or disruption of Nrf2 affects the development of colorectal adenoma. We found that the multiplicity of tumors was not affected by either disruption of Nrf2 or its genetic upregulation by Keap1 knockdown (Fig. 2a, b). Interestingly, the multiplicity of large (>2.5 mm diameter) tumors tended to be lower in female mice than in male mice, again with no significant differences among the genotypes (Fig. 2c, d). Overall tumor burden was also similar among the genotypes, and greater in male than female animals (Fig. 2e, f).

### Nrf2 activation does not affect the expression of pro-inflammatory cytokines in tumor or peri-tumoral tissue

**Fig. 3:**
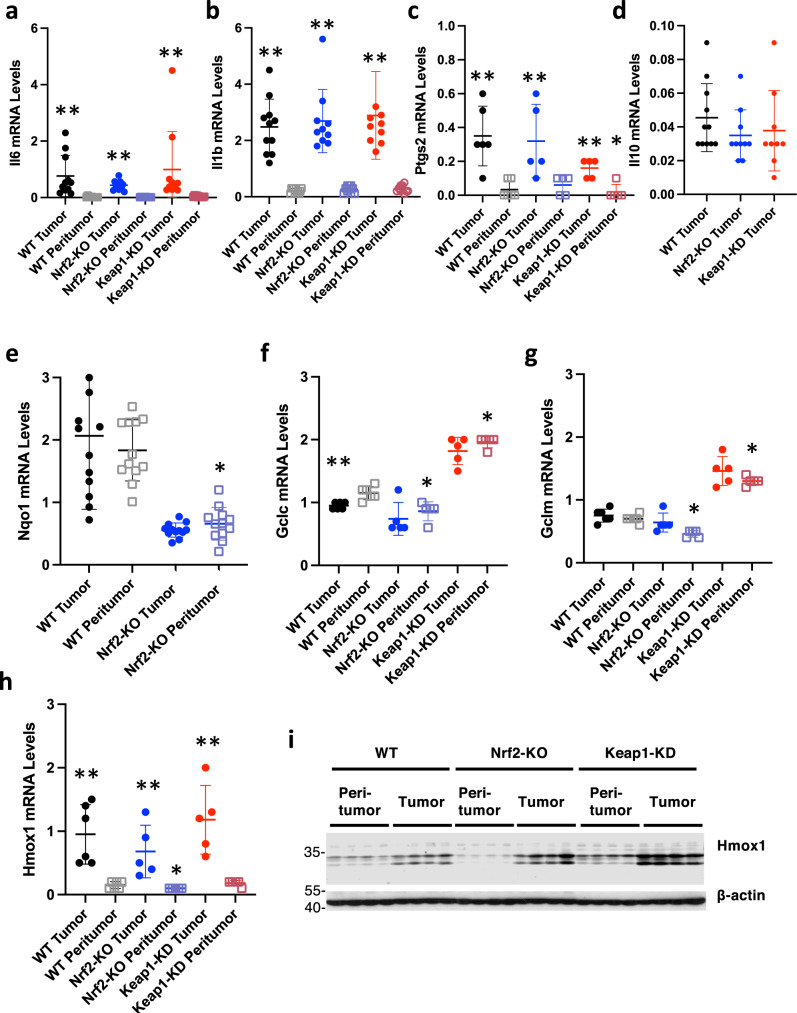
Nrf2 activation does not affect the expression of pro-inflammatory cytokines in tumor or peri-tumoral tissue. **a**–**c** mRNA levels for Il1β (**a**), Il6 (**b**) Ptgs2 (**c**), and Il10 (**d**) in colorectal tumors and peri-tumoral colon tissue of female Gstp^−/−^: Apc^Min/+^: Nrf2^+/+^: Keap1^+/+^ (WT), Gstp^−/−^: Apc^Min/+^: Nrf2^−/−^: Keap1^+/+^(Nrf2-KO), and Gstp^−/−^: Apc^Min/+^: Nrf2^+/+^: Keap1^flox/flox^ (Keap1-KD) mice (*n* = 5–11). Hprt1 was used as the reference gene. **e** mRNA levels for Nqo1 in tumor vs. peri-tumoral tissues in Gstp^−/−^: Apc^Min/+^: Nrf2^+/+^: Keap1^+/+^ (WT) and Gstp^−/−^: Apc^Min/+^: Nrf2^−/−^: Keap1^+/+^ (Nrf2-KO) mice (*n* = 12). Rplp0 was used as the reference gene. **f**–**h** mRNA levels for Gclc (**f**), Gclm (**g**), and Hmox1 (**h**) in tumor vs. peri-tumoral tissues in Gstp^−/−^: Apc^Min/+^: Nrf2^+/+^: Keap1^+/+^ (WT) and Gstp^−/−^: Apc^Min/+^: Nrf2^−/−^: Keap1^+/+^(Nrf2-KO) mice (*n* = 5–6). Rplp0 was used as the reference gene. **i** Protein levels for Hmox1 in tumor vs. peri-tumoral tissues in Gstp^−/−^: Apc^Min/+^: Nrf2^+/+^: Keap1^+/+^ (WT) and Gstp^−/−^: Apc^Min/+^: Nrf2^−/−^: Keap1^+/+^(Nrf2-KO) mice (*n* = 4). β-actin was used as loading control. *p < 0.05 vs. WT peri-tumor; **p < 0.05 tumor vs. peri-tumor.

In a subset of mice, we determined the expression of the pro-inflammatory cytokines Il6 and Il1β, and the pro-inflammatory enzyme Ptgs2. The mRNA levels for both cytokines were higher in tumor compared to peri-tumoral tissue, had high inter-tumor variability, and did not differ significantly among the genotypes (Fig. 3a, b). The expression of Ptgs2 was increased in the tumors (Fig. 3c), although this increase was smaller in tumors from Keap1-KD mice in comparison with WT or Nrf2-KO, consistent with our previous observations of a reciprocal relation between pharmacological Nrf2 activation and interferon-γ (IFNγ)-stimulated Ptgs2 transcription^5^. The expression of the anti-inflammatory cytokine Il10 in tumor tissue was not affected by either Nrf2 disruption or genetic activation (Fig. 3d). Notably, the mRNA levels for Nqo1 in tumor and peri-tumoral tissues in WT (i.e. Gstp^−/−^: Apc^Min/+^: Nrf2^+/+^: Keap1^+/+^) mice were highly variable among 12 randomly selected individual animals (Fig. 3e), suggesting no consistent changes in Nrf2-transcriptional activity during adenoma development. Although lower than in WT, as expected based on its regulation by Nrf2, the Nqo1 expression did not differ between tumor and peri-tumoral tissues of Nrf2-KO mice. Similarly, the mRNA levels for Gclc and Gclm, two other Nrf2-transcriptional targets encoding the catalytic and the modifier subunits, respectively, of glutamate-cysteine ligase, the enzyme catalyzing the rate-limiting step in the biosynthesis of glutathione (GSH), were similar in tumor compared to peri-tumoral tissue, although both were higher in tissues from Keap1-KD compared to WT mice (Fig. 3f, g), as expected^31^. Interestingly, however, the mRNA (Fig. 3h) and the protein (Fig. 3i) levels for heme oxygenase 1 (Hmox1), which is also partly regulated by Nrf2, were profoundly increased in tumor vs. peri-tumoral tissues. The protein levels of Hmox1 were lowest in Nrf2-KO and highest in Keap1-KD peri-tumoral tissues, as expected. By contrast, the increase in Hmox1 levels in the tumor tissues was higher in Nrf2-KO than in WT mice, indicating that transcription factors other than Nrf2 were responsible for it. Considering that the expression of Hmox1 is also regulated by the pro-inflammatory transcription factor nuclear factor κB (NFκB) and activator protein 1 (AP1)^32^, and that Hmox1 is known to be upregulated in inflamed colonic tissues^33^, we speculate that the enhanced Hmox1 expression in the tumors is at least in part due to the markedly enhanced inflammation in tumor compared to peri-tumoral tissue (Fig. 3a–c).

### Colorectal adenomas that form in Gstp^−/−^: Apc^Min/+^ mice are characterized by altered one-carbon metabolism

**Fig. 4:**
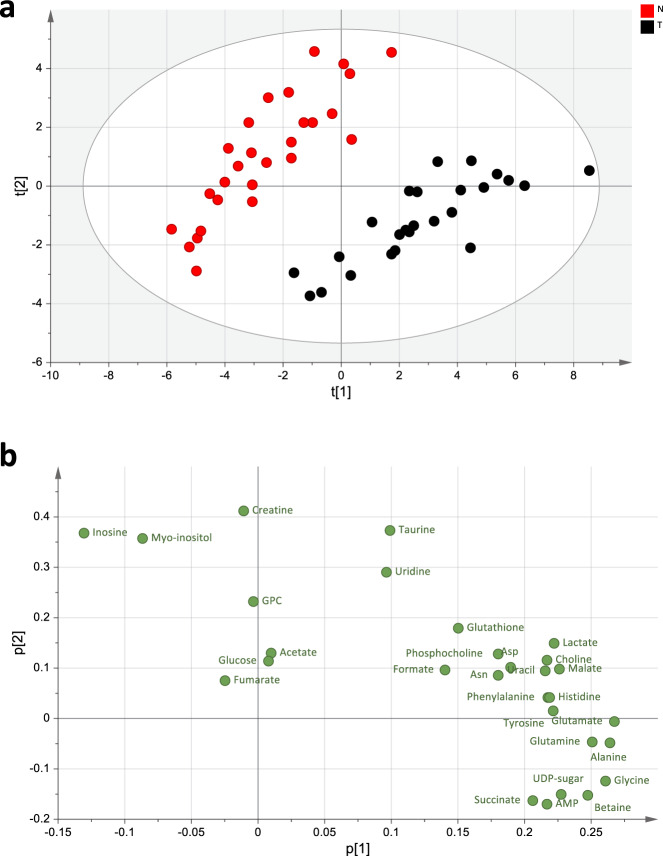
Tumor and peri-tumoral tissues have distinct metabolic states as revealed by NMR spectroscopy analysis. **a** Principal component analysis (PCA) score plot from NMR data for total fatty acids (*R*^2 ^= 75.9% and *Q*^2 ^= 41.7%). At least seven independent biological replicates of colon tissue from peri-tumorous (red) and tumorous (black) tissues were included. **b** Plot showing the individual metabolites driving the separation among the tissue types (loadings), with the variables (i.e., metabolites) represented in green circles (Asp = aspartate and Asn = asparagine).

**Fig. 5:**
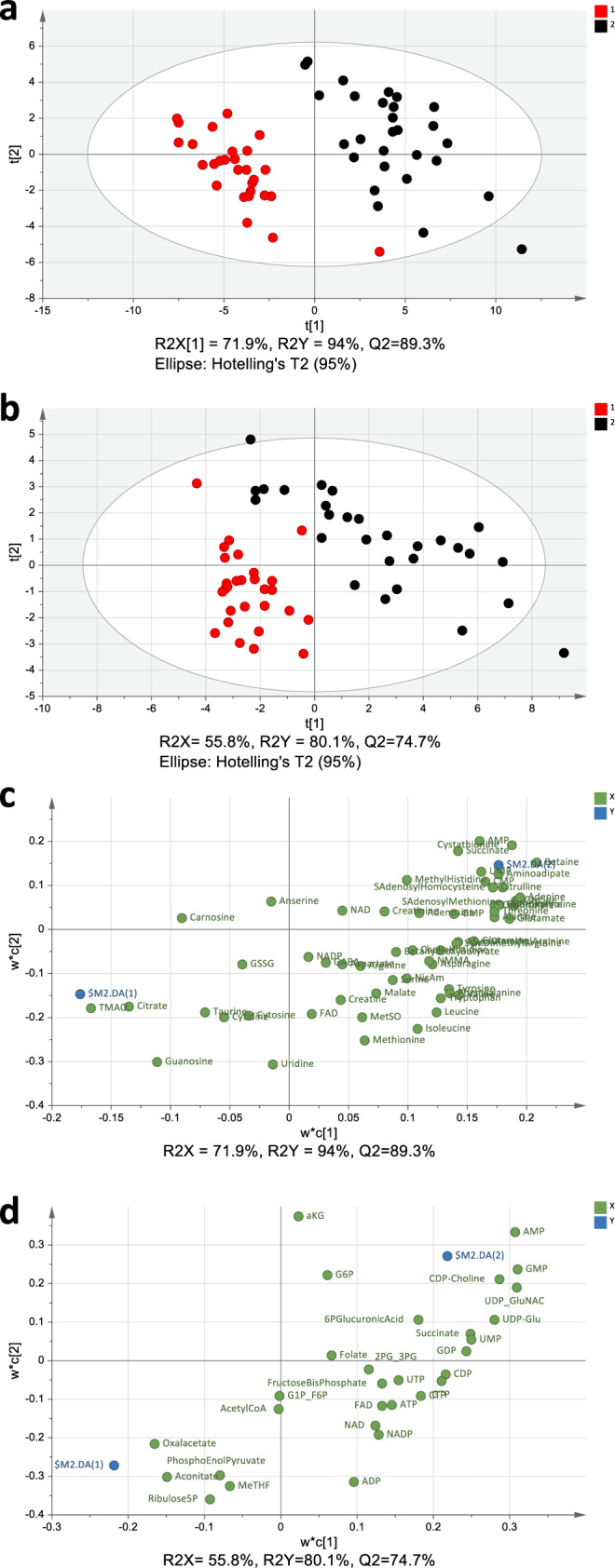
Tumor and peri-tumoral tissues have distinct metabolic states as revealed by LC-MS analysis. **a**, **b** Partial least square-discriminant analysis (PLS-DA) score plot from LC-MS data for aqueous metabolites, analyzed by**:** a C18pfp-based method (*R*^2^*X* = 71.9%, *R*^2^*Y* = 94% and *Q*^2 ^= 89.3%) (**a**); and a behamide-based method (*R*^2^*X* = 55.8%, *R*^2^*Y* = 80.1% and *Q*^2 ^= 74.7%) (**b**). In both cases, at least seven independent biological replicates of colon tissue from peri-tumorous (red) and tumorous (black) tissues were included. **c**, **d** Plots showing the individual metabolites driving the separation among the tissue types (loadings), for the C18pfp-based method (**c**) and the behamide-based method (**d**), with the variables (i.e., metabolites) represented in green circles, while the center for each genotype (peri-tumorous-DA(1), tumorous-DA(2)) is in blue.

**Fig. 6:**
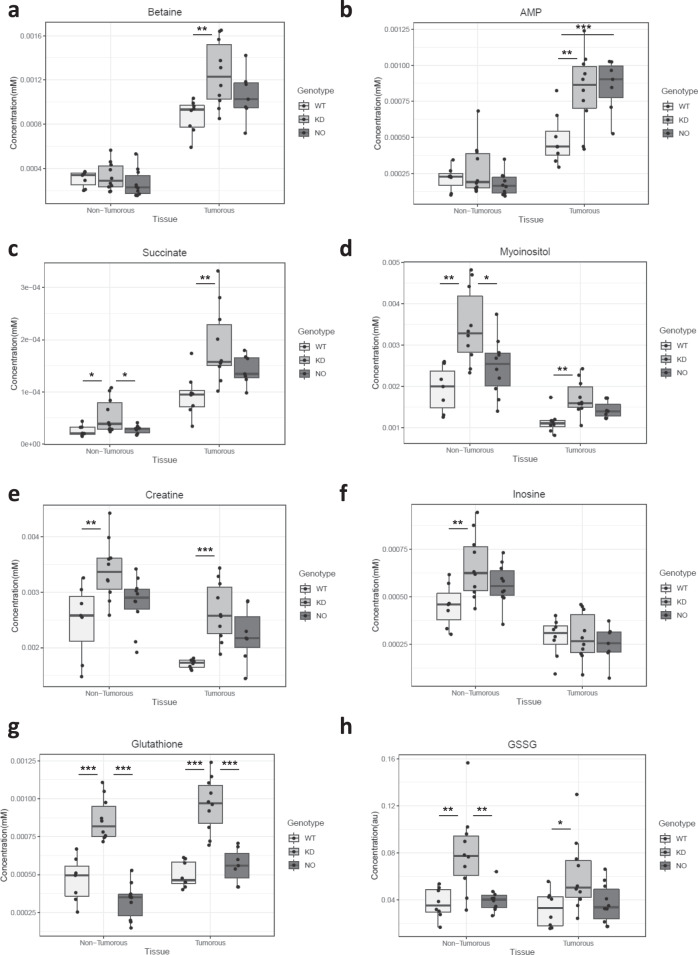
Colorectal adenomas have altered metabolite levels in comparison with matched peri-tumorous tissue. **a**–**f** Concentration of selected metabolites in peri-tumorous and tumorous tissues of wild-type (white bars), Keap1-KD (gray bars) and Nrf2-KO (black bars) mice as measured by NMR. (**a**) betaine, (**b**) AMP, (**c**) succinate, (**d**) myo-inositol, (**e**) creatine, (**f**) inosine. **g**, **h** Concentration of glutathione (GSH) (**g**) and GSSG (**h**) in peri-tumorous and tumorous tissues of wild-type (white bars), Keap1-KD (black bars) and Nrf2-KO (gray bars) as measured by NMR. *0.05 > *p* > 0.01, ** 0.01 > *p* > 0.001, ***p < 0.001, using ANOVA followed by Tukey post-hoc test for statistical significance.

By use of metabolomics, combining nuclear magnetic resonance (NMR) spectroscopy and two complementary liquid chromatography mass spectrometry (LC-MS) approaches, we examined if we could identify a metabolic signature induced by the tumor, irrespectively of the genotype. Multivariate analysis of both the NMR spectroscopy (Fig. 4a, b) and the LC-MS data (Fig. 5a–d) showed a clear separation between tumors and peri-tumoral tissues, indicating distinct metabolic states, with no apparent subgrouping related to the genotype. The NMR spectroscopy analysis showed significantly higher levels of betaine, AMP, succinate, alanine, glutamate, glutamine, glycine, aspartate, and malate (Fig. 6a–c and Supplementary Fig. 1) in the tumors compared to peri-tumoral tissues, and lower levels of myo-inositol, creatine and inosine (Fig. 6d, e). The increase in betaine (Fig. 6a) was particularly intriguing, due to its role in methylation. The LC-MS analyses confirmed the higher AMP levels in tumors, and further showed increases in the ribonucleotides GMP, CMP and UMP (Supplementary Fig. 1c and Supplementary Fig. 2). This analysis also confirmed the increased glutamate and glycine levels, and additionally showed that the levels of proline, hydoxyproline and CDP-choline were also increased (Supplementary Fig. 1d, e). Furthermore, in addition to confirming higher levels of betaine (Supplementary Fig. 1f), this analysis revealed increases in choline, *S*-adenosyl methionine (SAM), *S*-adenosyl homocysteine (SAH) (Supplementary Fig. 1f, g), and cystathionine (Supplementary Fig. 1e). Together, these changes indicate that one-carbon metabolism is altered in the colorectal adenomas from Gstp^−/−^: Apc^Min/+^ mice.

### Knockdown of Keap1, but not disruption of Nrf2, enhances the metabolic alterations in colorectal adenomas from Gstp^−/−^: Apc^Min/+^ mice

We next focused our analysis on differences among the genotypes within the same type of tissue. Both the NMR spectroscopy and the LC-MS analyses showed that the levels of glutathione were higher in both tumors and peri-tumoral tissues from Keap1-KD mice, in comparison with their WT and Nrf2-KO counterparts (Fig. 6g, h), in close agreement with the higher expression levels of Gclc and Gclm (Fig. 3f, g). Glutathione levels tend towards an increase, but this increase is not statistically significant between peri-tumor and tumor samples (*p* = 0.059), if the genotype factor is ignored. However, plotting its levels in all three genotypes in both types of tissue shows the different behavior in each case. Significantly different amounts of glutathione were found in both the peri-tumoral tissues (ANOVA *p* = 1.37E−8) and the tumors (ANOVA *p* = 5.42E−7), with a particular increase in the Keap1-KD genotype in both cases. This plot also shows that Nrf2-KO is the only genotype where glutathione increases in the tumors.

**Fig. 7:**
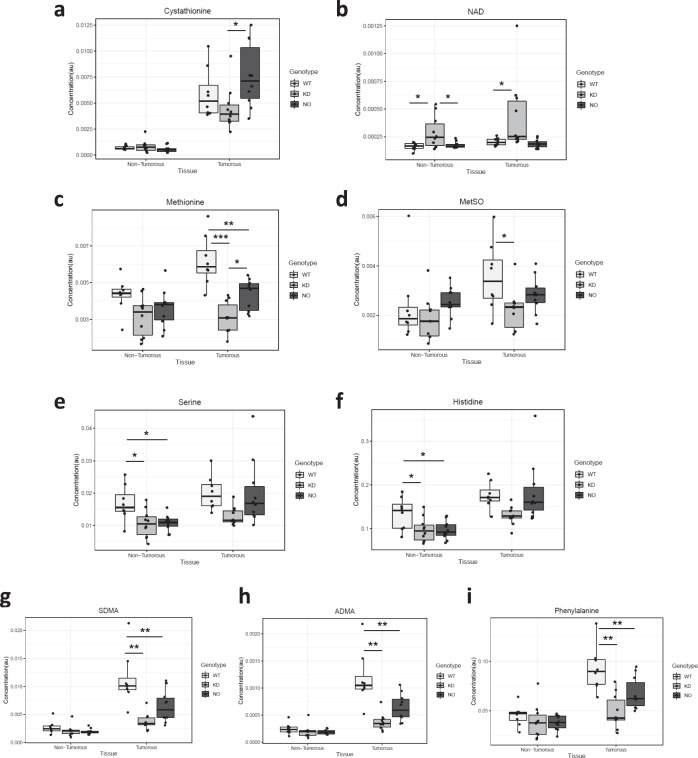
Knockdown of Keap1 enhances the metabolic alterations in colorectal adenomas. Concentration of selected metabolites in peri-tumorous and tumorous tissues of wild-type (white bars), Keap1-KD (gray bars) and Nrf2-KO (black bars) mice as measured by LC-MS using the C18pfp-based method. (**a**) cystathione, (**b**) NAD, (**c**) methionine, (**d**) methionine sulfoxide (MetSO), (**e**) serine, (**f**) histidine, (**g**) symmetric dimethylarginine (SDMA), (**h**) asymmetric dimethylarginine (ADMA), (**i**) phenylalanine. *0.05 > *p* > 0.01, **0.01 > *p* > 0.001, ***p < 0.001, using ANOVA followed by Tukey post-hoc test for statistical significance.

In general, the differences in metabolites between WT and Keap1-KD were greater than the differences between WT and Nrf2-KO genotypes in both tumor and peri-tumoral tissues. Thus, compared to WT, the levels of myo-inositol (Fig. 6d), betaine (Fig. 6a), AMP (Fig. 6b), succinate (Fig. 6c), creatine (Fig. 6e) and NAD (Fig. 7b) were higher in tumor tissue of Keap1-KD mice, whereas the levels of methionine (Fig. 7c) and methionine sulfoxide (Fig. 7d) were lower. By contrast, only the levels of AMP (Fig. 6b) and methionine (Fig. 7c) differed between WT and Nrf2-KO tumors. In peri-tumoral tissue, the levels of myo-inositol (Fig. 6d), succinate (Fig. 6c), creatine (Fig. 6e), NAD (Fig. 7b), and inosine (Fig. 6f) were higher in Keap1-KD compared to WT mice, whereas the levels of serine (Fig. 7e) and histidine (Fig. 7f) were lower. Interestingly, the levels of cystathione were increased in the tumors, and although still increased in tumor compared to peri-tumoral tissue, were lower in tumors from Keap1-KD and higher in tumors from Nrf2-KO mice (Fig. 7a). Finally, symmetric dimethylarginine (SDMA) (Fig. 7g), asymmetric dimethylarginine (ADMA) (Fig. 7h) and phenylalanine (Fig. 7i) were markedly increased in tumor compared to peri-tumoral tissue, but these increases were much less pronounced in tumors from either Keap1-KD or Nrf2-KO mice.

**Fig. 8:**
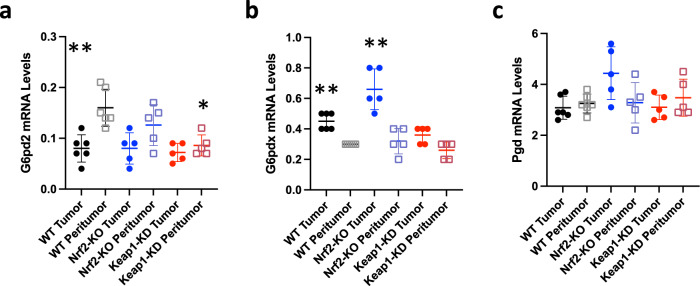
The expression of G6pdx, G6pd2 and Pgd is not coordinately altered in colorectal adenomas. **a**–**c** mRNA levels for G6pd2 (**a**), G6pdx (**b**) and Pgd (**c**) in tumor vs. peri-tumoral tissues in Gstp^−/−^: Apc^Min/+^: Nrf2^+/+^: Keap1^+/+^ (WT) and Gstp^−/−^: Apc^Min/+^: Nrf2^−/−^: Keap1^+/+^(Nrf2-KO) mice (*n* = 5–6). Rplp0 was used as the reference gene. *p < 0.05 vs. WT peri-tumor; **p < 0.05 tumor vs. peri-tumor.

Overall, most differences in metabolites between tumor and peri-tumoral tissues were enhanced in Keap1-KD mice; however, the magnitude of this enhancement was much more modest in comparison with the magnitude of the differences between tumor and peri-tumoral tissues. This observation is further supported by the fact that the mRNA levels for glucose-6-phosphate dehydrogenase (G6pdx and G6pd2) (Fig. 8a, b) and the pentose phosphate pathway enzyme phosphogluconate dehydrogenase (Pgd) (Fig. 8c), which increase upon Nrf2 activation in rapidly proliferating cells^18^, were not different among the Nrf2/Keap1 genotypes. Interestingly, the expression of G6pdx was increased in tumor in comparison with peri-tumoral tissues, whereas that of G6pd2 was decreased, and the mRNA levels for Pgd were unchanged. Together, these findings suggest that in this model, the enhanced metabolic changes conferred by the Keap1 knockdown may have negligible contribution to the carcinogenesis process, a conclusion also supported by the fact that neither Keap1 knockdown nor disruption of Nrf2 affected colorectal adenoma formation in Gstp^−/−^: Apc^Min/+^ mice (Fig. 2).

### Chronic pharmacological activation of Nrf2 by TBE-31 does not affect colorectal adenoma formation in Gstp^−/−^: Apc^Min/+^ mice

**Fig. 9:**
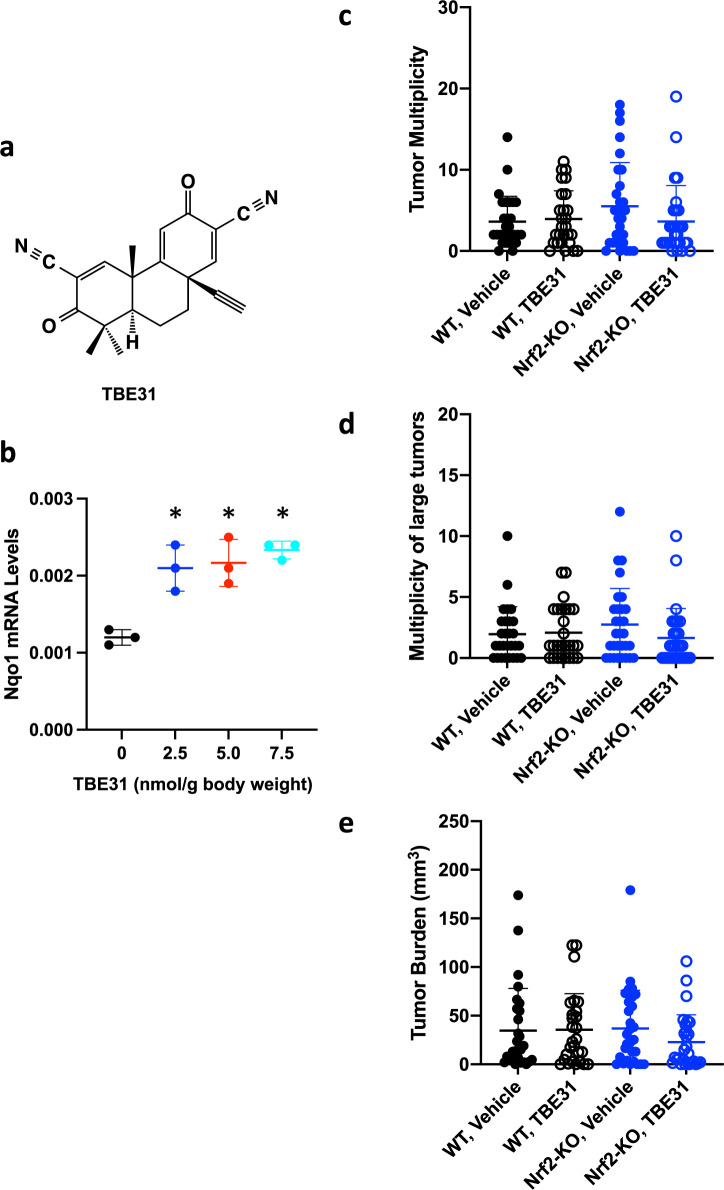
Chronic pharmacological activation of Nrf2 by TBE-31 does not affect colorectal adenoma formation in Gstp^−/−^: Apc^Min/+^ mice. **a** Chemical structure of TBE-31. **b** mRNA levels for Nqo1 in colons from Gstp^−/−^: Apc^Min/+^: Nrf2^+/+^: Keap1^+/+^ (WT) mice (*n* = 3–4) that had been treated with vehicle (0.7% DMSO in corn oil) or the indicated doses of TBE-31, *per os*, 3 days per week for 1 week, 72h-, 48h- and 48h-apart; colon tissue was harvested 24 h after the last dose. Actb was used as the reference gene. *p < 0.05 vs. vehicle-treated. **c**–**e** Tumor multiplicity (**c**, **d**) and burden (**e**) in colons of 20-week-old Gstp^−/−^: Apc^Min/+:^ Nrf2^+/+^: Keap1^+/+^ (WT, black circles) and Gstp^−/−^: Apc^Min/+:^ Nrf2^−/−^: Keap1^+/+(^Nrf2-KO, blue circles) mice (*n* = 27–30) that had been treated *per os* with vehicle (0.7% v/v DMSO in corn oil, closed circles) or TBE-31 (5 nmol/g body weight, 3 days per week for 12 weeks, starting at week 8 of age, open circles).

We used the potent tricyclic cyanoenone TBE-31 (Fig. 9a) to activate Nrf2 pharmacologically. The mRNA levels for Nqo1 (Fig. 9b), were increased by oral administration of TBE-31, confirming Nrf2 activation in the colons of Gstp^−/−^: Apc^Min/+^ mice.

In full agreement with the previous experiment (Fig. 2), there was no significant difference in tumor multiplicity (Fig. 9c, d) or burden (Fig. 9e) between WT and Nrf2-KO mice, confirming that the absence of functional Nrf2 neither accelerates nor inhibits tumor development in this model. Consistent with the lack of effect of genetic Nrf2 activation by Keap1 knockdown, chronic intervention with TBE-31 (5 nmol/g body weight, orally, 3 days per week for 12 weeks, starting at week 8 of age) did not affect tumor development in Gstp^+−/+−^: Apc^Min/+^ mice.

### Genetic activation or disruption of Nrf2 does not affect colorectal adenoma formation in Gstp^−/−^: Apc^Min/+^ mice fed high-fat diet

**Fig. 10:**
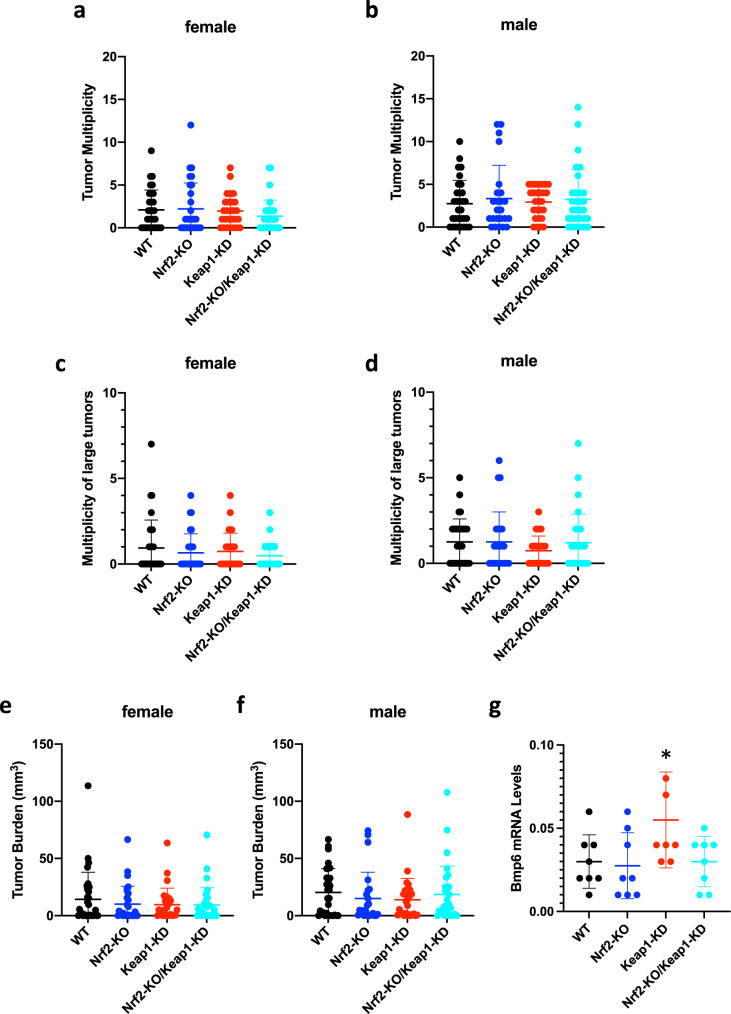
Genetic activation or disruption of Nrf2 does not affect colorectal adenoma formation in Gstp^−/−^: Apc^Min/+^ mice fed high-fat diet. Tumor multiplicity (**a**–**d**) and burden (**e**, **f**) in colons of 15-week-old Gstp^−/−^: Apc^Min/+^: Nrf2^+/+^: Keap1^+/+^ (WT, black circles), Gstp^−/−^: Apc^Min/+^: Nrf2^−/−^: Keap1^+/+^(Nrf2-KO, blue circles), Gstp^−/−^: Apc^Min/+^: Nrf2^+/+^: Keap1^flox/flox^ (Keap1-KD, red circles), and Gstp^−/−^: Apc^Min/+^: Nrf2^−/−^: Keap1^flox/flox^ (Nrf2-KO/Keap1-KD, cyan circles) mice (*n* = 53–62) that had been fed high-fat diet for 9 weeks. **g** mRNA levels for Bmp6 in livers of 15-week-old Gstp^−/−^: Apc^Min/+^: Nrf2^+/+^: Keap1^+/+^ (WT, black circles), Gstp^−/−^: Apc^Min/+^: Nrf2^−/−^: Keap1^+/+^(Nrf2-KO, blue circles), Gstp^−/−^: Apc^Min/+^: Nrf2^+/+^: Keap1^flox/flox^ (Keap1-KD, red circles), and Gstp^−/−^: Apc^Min/+^: Nrf2^−/−^: Keap1^flox/flox^ (Nrf2-KO/Keap1-KD, cyan circles) mice (*n* = 8) that had been fed high-fat diet for 9 weeks. Rplp0 was used as the reference gene. *p < 0.05 vs. WT.

The combination of high-fat diet and Apc truncation has been shown to alter the bile acid profile, antagonizing the function of intestinal farnesoid X receptor (FXR) to drive malignant transformation in cancer stem cells^34^. Thus, we asked if genetic alterations in Nrf2 may affect tumor development in mice fed high-fat diet, which is increasingly more common worldwide. To this end, starting at week 6 of age, the animals were fed diet delivering 60% of calories from fat for 9 weeks, and tumor development was assessed at week 15. Although Keap1 is the main negative regulator of Nrf2, the activity of the transcription factor in cancer is also affected by several other proteins^35^. Similarly, in addition to Nrf2, Keap1 regulates the function of other cellular proteins^36^. Thus, in addition to the three genotypes (i.e. WT, Nrf2-KO and Keap1-KD), for this experiment we also generated a fourth category of mice combining Nrf2 deficiency and Keap1 downregulation, namely Gstp^−/−^: Apc^Min/+^: Nrf2^−/−^: Keap1^flox/flox^, to which we refer as Nrf2-KO/Keap1-KD double-transgenic mice. Consistent with the carcinogenesis experiments described above, all mice were Gstp^−/−^: Apc^Min/+^. Once again, we found that tumor multiplicity tended to be higher in male than in female mice, and that there were no significant differences in the multiplicity (Fig. 10a–d) or burden (Fig. 10e, f) of colorectal tumors among any of the four genotypes, in close agreement with the previous experiments in mice fed standard diet (Figs. 2, 9c–e). This experiment further confirmed the conclusion that disruption of Nrf2 or its constitutive upregulation by either genetic or pharmacological means, does not affect colorectal adenoma development in this mouse model even under conditions of high-fat diet.

Notably, it was necessary to terminate the high-fat diet experiment earlier than the experiments on standard diet, when the animals were 15- rather than 20-week old, and we had to enroll many more mice as a number of them had to be sacrificed before the 15-week endpoint. This necessity was related to concerns for animal welfare, the close monitoring of which showed that many of the mice on high-fat diet developed anemia earlier than those on standard diet. Indeed, it has been shown that C57BL/6 mice fed high-fat diet for 8 weeks develop iron deficiency due to diminished intestinal iron uptake^37^. Curiously, we found that anemia was more prevalent in Keap1-KD animals than in any of the other three genotypes (*p* < 0.001 for Keap1-KD vs. WT; *p* = 0.012 for Keap1-KD vs. Nrf2-KO; *p* = 0.005 for Keap1-KD vs. Nrf2-KO/Keap1-KD). Thus, the percentage of anemic mice within each genotype was: WT: 4.8% (3/62; 3 males and 0 females), Nrf2-KO: 11.7% (7/60; 5 males and 2 females), Keap1-KD: 27.4% (17/62; 9 males and 8 females), and Nrf2-KO/Keap1-KD: 9.8% (6/61; 4 males and 2 females). We hypothesized that the increased anemia in the Keap1-KD mice was at least in part caused by the documented Nrf2-dependent transcription of bone morphogenetic protein 6 (Bmp6) in liver sinusoidal endothelial cells^38^. This is because Bmp6 is known to regulate the synthesis of the liver hormone hepcidin, reducing serum iron levels^39^. Therefore, we measured the hepatic mRNA levels for Bmp6, and found them to be higher in livers of Keap1-KD mice compared to their WT or Nrf2-KO counterparts (Fig. 10g). Consistent with Nrf2 dependence, the hepatic expression levels of Bmp6 did not differ from WT in Keap1-KD mice with disrupted Nrf2 (i.e. Nrf2-KO/Keap1-KD double transgenic) mice. We attribute the small magnitude of hepatic Bmp6 increase in Keap1-KD mice to the fact that the liver sinusoidal endothelial cells represent ~15% of the total liver cell population, and only 3% of the liver volume^40^. Alternatively, it is possible that other transcription factors attenuate the Nrf2-mediated increase in Bmp6 transcription, such as HIF1α, which is upregulated by iron deficiency^41^, and has been shown to downregulate the expression of Bmp6^42^ as well as to counteract Nrf2-dependent gene expression^43^.

## Discussion

Over the years since its discovery, Nrf2 has been attributed both tumor-suppressing and tumor-promoting effects, and has been linked to the hallmarks of cancer^44^. Activation of Nrf2 is commonly observed in established human tumors^45^, raising concerns over the current drug development and clinical use of pharmacological Nrf2 activators. In this study, we addressed this concern by use of a mouse model of colorectal cancer initiated by a truncation of the *Apc* gene. Inactivating mutations of both alleles of the tumor suppressor *APC* represent one of the earliest events in the colorectal carcinogenesis in humans^46^. Our results show that neither genetic (by Keap1 knockdown) nor pharmacological (by a potent activator, the cyanoenone TBE-31) Nrf2 activation affects adenoma development at the early stages. However, Nrf2 is upregulated in human colorectal tumors and correlates with poor patient prognosis^22^, and thus it is probable that Nrf2 activation occurs later in the carcinogenesis process, such as during transition from adenoma to adenocarcinoma, supporting tumor growth. Further studies are needed to examine this possibility, which would require the use of a different animal model. One restriction of the model used in our study is that, in keeping with animal welfare regulations, it is not possible to maintain the experimental mice beyond the adenoma stage of the tumors, which limits its clinical relevance to the early stages of the carcinogenesis process.

TBE-31 is a cyanoenone, which shares the same electrophilic centers with bardoxolone methyl (CDDO-Me) and RTA-408, two pentacyclic cyanoenone triterpenoids, which are currently in advanced clinical trials for a number of disease indications, including Alport syndrome, chronic kidney disease associated with type 2 diabetes mellitus, liver disease, Friedreich’s ataxia^4,47^, and in patients hospitalized with confirmed COVID-19 (NCT04494646). The concern that pharmacological Nrf2 activation may promote the development of early neoplastic lesions is particularly relevant to colorectal cancer, because it is the third most common cancer globally in both men and women, with high mortality rates^48^, and because orally-administered pharmacological Nrf2 activators are in direct contact with the gastrointestinal tract epithelium. The findings from our study suggest that it is unlikely that pharmacological Nrf2 activators that are being developed for clinical use will promote the initiation of colorectal cancer.

Using LC-MS analysis of metabolites in colon tissue extracts, we recently found that the levels of glucose 6-phosphate and fructose 6-phosphate, metabolites involved in the initial steps of glycolysis, were higher in colons of Keap1-KD mice in comparison with their WT counterparts, whereas dihydroxyacetone phosphate and glyceraldehyde 3-phosphate, which are involved in the later steps of glycolysis, were lower^30^. Additionally, gas chromatography-MS (GC-MS) analysis showed that the levels of saturated and mono-unsaturated fatty acids were lower in Keap1-KD mice, whereas the levels of polyunsaturated fatty acids were higher. Together, these findings indicate that knockdown of Keap1, and the consequent activation of Nrf2, affect multiple aspects of intermediary metabolism in the healthy colon. Here, we show that the differences in metabolites between tumor and peri-tumoral colon tissues of Gstp^−/−^: Apc^Min/+^ mice are enhanced by Keap1 knockdown. Nonetheless, the magnitude of this enhancement is modest compared to the magnitude of the difference in the metabolic state between tumor and peri-tumoral tissues. These observations, together with the finding that neither genetic Keap1 knockdown nor its pharmacological inactivation by TBE-31 affects adenoma formation, lead us to conclude that in this model, the enhanced metabolic changes conferred by the Keap1 knockdown do not substantially contribute to the early stages of the colorectal carcinogenesis process.

It is noteworthy that tumor multiplicity, particularly that of large (>2.5 mm diameter) tumors, and burden are consistently higher in male than in female mice in all of our experiments. This finding is in agreement with the increased prevalence of colorectal cancer in males in human populations^49^ and the enhanced adenoma multiplicity in the colon of Apc^Min/+^ mice (that are wild-type for Gstp) and rats carrying a nonsense mutation in the Apc gene, termed Polyposis in the rat colon (Apc^Pirc/+^), as well as in rat models of colorectal carcinogenesis induced by the chemical carcinogens 1,2-dimethylhydrazine^50^ or azoxymethane^51^. Detailed studies by Amos-Landgraf et al^51^. have found that neither ovariectomy nor hormone replacement affected the prevalence of adenomas in female Apc^Pirc/+^ rats, whereas orchidectomy reduced adenoma development in their male counterparts, and supplementation with testosterone reversed this effect. Thus, as in the above discussed colorectal cancer models, the observed sexual dimorphism in multiplicity of colonic tumors in our model is more likely due to tumor promotion by male hormones rather than protection by female hormones^51^.

Cheung et al.^27^ have reported increased inflammation and intestinal carcinogenesis in Gstp^+/+^: Apc^Min/+^: Nrf2^−/−^: Keap1^+/+^ mice^52^. In our model, unbiased pathway enrichment analysis following mRNA profiling, as well as additional supportive biochemical analyses, revealed that the absence of Gstp promotes a state of enhanced inflammation, accompanied by a 6-fold increase in colon adenoma incidence, and a 50-fold increase in colorectal adenoma multiplicity. The present study confirmed the profound increase in pro-inflammatory markers (Il6, Il1β, and Ptgs2) in tumor compared to peri-tumoral tissue in mice of all genotypes (Fig. 3a–c). Importantly however, Nrf2 disruption did not increase inflammation further (Fig. 3a–c). Together, these studies illustrate the critical importance of inflammation in colorectal adenoma formation, consistent with the tumor-suppressive effects of anti-inflammatory drugs, such as celecoxib^53,54^ and aspirin^55,56^ in Apc^Min/+^ mice, and the epidemiological data showing association of regular aspirin use with reduced incidence of colorectal cancer in humans^57^.

Of note, quantitative proteomic analysis of livers from Gstp^−/−^ mice, which were used to obtain the Gstp^−/−^: Apc^Min/+^ mice, showed similar overall protein *S*-glutathionylation profiles to those of their Gstp^+/+^ counterparts, indicating that whereas Gstp may be involved in catalyzing *S-*glutathionylation of some proteins in response to oxidative stress, including Keap1^58^, its role in global *S*-glutathionylation is limited^59^. Nonetheless, the absence of Gstp is an important aspect of our mouse models because *S*-glutathionylation of Keap1 may lead to Nrf2 activation^58^.

An interesting corollary of this study is the observation that a number of metabolites involved in one-carbon metabolism (comprising the folate and methionine cycles) are altered in the colorectal adenomas from Gstp^−/−^: Apc^Min/+^ mice. The enhanced effect of the Keap1 knockdown is consistent with the importance of Keap1/Nrf2 for mitochondrial health^60,61^, which in turn supports one-carbon metabolism^62^. One-carbon metabolism and SAM have critical roles in multiple cellular processes, including generation of precursors to nucleotide biosynthesis and one-carbon units for DNA- and histone-methylation reactions, and can drive tumorigenesis^63^. Crucially, because all tumors in this model are adenomas^27^, our findings strongly suggest that alterations in one-carbon metabolism occur early in the colorectal carcinogenesis process, and together with changes in expression/activity of the enzymes that catalyze their formation, these metabolic alterations allow for the well-documented aberrations in DNA and histone methylation in colorectal tumors from mice and humans^48^. Curiously, Khor et al.^64^ have reported promoter hypermethylation of *NFE2L2*, the gene encoding Nrf2, and a trend for a decrease in the intensity and percentage of Nrf2-positive cells in advanced-stage prostate cancer compared to normal prostate tissue. Conversely, Hanada et al.^65^ have shown hypermethylation of the Keap1 promoter, leading to decreased expression and increased nuclear Nrf2 and downstream Nrf2-target gene expression in colorectal cancer. Thus, it is possible that the observed increase in one-carbon metabolism in our mouse models may additionally affect Keap1 and/or Nrf2 expression via epigenetic mechanisms.

Another interesting observation from this study is the increased prevalence of anemia in Keap1-KD animals fed high-fat diet than in any of the other three genotypes. The fact that this phenotype is more common in Keap1-KD than in Nrf2-KO/Keap1-KD double-transgenic mice suggests that it is related to Nrf2 activation rather than any other process that might be influenced by the downregulation of Keap1. We found that the mRNA levels for Bmp6, which controls the synthesis of the liver hormone hepcidin that in turn lowers serum iron levels^39^, are higher in livers of Keap1-KD, but importantly not in Nrf2-KO/Keap1-KD mice, in comparison with their WT or Nrf2-KO counterparts (Fig. 10g), consistent with Nrf2-dependent upregulation of hepcidin in mice fed high-fat plus iron^66^ or polyphenol-rich^67^ diets. In the small intestine, hepcidin is the predominant negative regulator of iron absorption by enterocytes^68^. As mentioned in Results, additionally and independently of the levels of hepcidin, high-fat diet feeding causes iron deficiency due to diminished intestinal iron uptake^37^. Thus, the increased hepcidin levels may exacerbate the diminished intestinal iron uptake due to the high-fat diet by limiting dietary iron absorption by enterocytes, ultimately causing anemia.

## Methods

### Animals

All mouse experiments were performed in accordance with the regulations described in the UK Animals (Scientific Procedures) Act 1986 following approval by the Welfare and Ethical use of Animals Committee of the University of Dundee. Experimental design was undertaken in line the 3Rs principles of replacement, reduction, and refinement (www.nc3rs.org.uk). Mice were bred and maintained at the Medical School Resource Unit of the University of Dundee, with free access to water and food (pelleted RM1 diet from SDS Ltd., Witham, Essex, UK), on a 12-h light/ 12-h dark cycle, 35% humidity. Gstp^−/−^: Apc^Min/+^: Nrf2^−/−^: Keap1^+/+^ and Gstp^−/−^: Apc^Min/+^: Nrf2^+/+^: Keap1^flox/flox^ mice were generated by crossing Gstp^−/−^: Apc^Min/+^ mice with Nrf2^−/−^ or Keap1^flox/flox^ mice, respectively, all on the C57BL/6 genetic background. Both male and female mice were used in the carcinogenesis experiments.

For administration to animals, (±)-TBE-31, synthesized as described^7,69^, was dissolved in DMSO (vehicle) and diluted (1:140) in corn oil to achieve the appropriate final concentrations indicated in the text and figures. The high-fat diet was RM AFE 60% FAT, 20% CP, 20% CHO (M) (SDS Ltd., Code 824054).

### Colorectal carcinogenesis

A scoring system was developed to monitor animal welfare and signs of intestinal neoplasia, including occult blood in faeces, rectal bleeding, stool consistency, anemia (by pale feet), rectal prolapse and weight loss. Mice were euthanized at defined time-points indicated in the text and figures, or when the scoring reached a set value. The large intestine was removed, flushed with PBS, placed on ice, cut longitudinally, the tumors counted, and their height, length, and width measured. Tumor volume was calculated according to a formula for the volume of a sphere, using the average of the three dimensions as the diameter. The tumors and peri-tumor tissue were then excised, snap-frozen in liquid N_2_ and stored at −80 °C till further analysis.

### Real-time quantitative PCR

Total RNA was extracted from mouse colon and tumor tissue using RNeasy Kit (Qiagen Ltd.), and 500 ng of total RNA was reverse-transcribed into cDNA using Omniscript RT Kit (Qiagen Ltd.). Real-time PCR was carried out on Applied Biosystems QuantStudio™ 5 Real-Time PCR System. The TaqMan data for the mRNA species were normalized using mouse ribosomal protein lateral stalk subunit P0 (Rplp0), hypoxanthine phosphoribosyltransferase 1 (Hprt1) and actin-beta (Actb) as internal controls. The TaqMan^TM^ Gene Expression Assay IDs (Thermo) used were: Mm00607939_s1 (Actb); Mm01332882_m1 (Bmp6); Mm00516005_m1 (Hmox1); Mm03024075_m1 (Hprt1); Mm00658204_s1 (G6pd2); Mm04260097_m1 (G6pdx); Mm00802655_m1 (Gclc); Mm00514996_m1 (Gclm); Mm04207463_m1 (GstA1/2); Mm00494798_m1 (GstA3); Mm00833915_g1 (GstM1); Mm00434228_m1 (Il1b); Mm00446190_m1 (Il6); Mm00439614_m1 (Il10); Mm01253561_m1 (Nqo1); Mm00503037_m1 (Pgd); Mm00478374_m1 (Ptgs2); Mm00725448_s1 (Rplp0).

### Immunoblotting

Frozen tissues (15-40 µg) were disrupted by coarse grinding in liquid N_2_, followed by homogenization for 40 sec in 10 volumes (µl per mg tissue) ice-cold RIPA buffer (50 mM Tris-HCl, pH 8.0; 150 mM sodium chloride; 1.0% NP-40; 0.5% sodium deoxycholate; 0.1% sodium dodecyl sulfate) supplemented with 1.4 × complete EDTA-free protease inhibitors cocktail (Roche). Homogenates were clarified by centrifugation for 10 min at 17,000 × *g* at 4 °C, protein concentrations in the supernatants were determined by the bicinchoninic acid (BCA) assay (Thermo) and adjusted to the same protein concentration in all samples using RIPA buffer. Samples were then mixed with NuPAGE LDS sample buffer (Thermo) and NuPAGE sample reducing agent (Thermo), heated for 10 min at 70 °C, and loaded at 20 µg/lane onto pre-cast gradient (4–12%) Tris-Glycine gels (Thermo). Proteins were resolved by electrophoresis and transferred onto 0.45-µm nitrocellulose membranes (Amersham Biosciences) at 100 V for 45 min at 4 °C. Membranes were blocked in 5% milk in PBST (0.1% Tween-20) for 45 min, on a rocker (60-70 rpm), at room temperature, and then incubated with the primary antibodies at 4 °C on a rocker overnight. Dilutions of all primary and secondary antibodies were in 5% milk in PBST (0.1% Tween-20). The following antibodies were used: rabbit polyclonal anti-Hmox1, 1:2,000, Abcam; mouse monoclonal anti-β-actin, 1:5,000. The uncropped/unedited scans of the immunoblots are shown in Supplementary Fig. 3.

### Metabolomics

Metabolites were extracted using the methanol/chloroform/water (2:2:1; v/v) method^70,71^. Briefly, 50 mg of wet weight tissue were mixed with 600 µl of CH_3_OH/CHCl_3_ (2:1; v/v), and the samples were homogenized with a Tissuelyser (Qiagen, UK) for 5 min at a frequency of 20/s and sonicated for 15 min. Water and chloroform (200 μl of each) were added to the samples before centrifugation at 13,300 rpm for 20 min. The resulting aqueous and organic phases were separated from the protein pellets. The extraction procedure was repeated on the remaining protein pellets as part of a double extraction procedure. Both organic and aqueous phases were collected and evaporated to dryness. The dried samples were stored at −80 °C until further analysis.

### NMR analysis of aqueous extracts

The dried aqueous fractions were rehydrated in 600 μl D_2_O, containing 0.05 mM sodium-3-(tri-methylsilyl)−2,2,3,3-tetradeuteriopropionate (TSP) (Cambridge Isotope Laboratories, MA, USA) as an internal standard. The samples were analyzed using an AVANCE II + NMR spectrometer operating at 500.13 MHz for the ^1^H frequency and 125.721 MHz for the ^13^C frequency (Bruker, Germany) using a 5 mm TXI probe. The instrument was equipped with TopSpin 3.2. Spectra were collected using a solvent suppression pulse sequence based on a one-dimensional nuclear Overhauser effect spectroscopy (NOESY) pulse sequence to saturate the residual ^1^H water signal (relaxation delay = 2 s, t1 increment = 3 µs, mixing time = 150 ms, solvent presaturation applied during the relaxation time and the mixing time). One hundred and twenty-eight transients were collected into 16 K data points over a spectral width of 12 ppm at 27 °C. Assignment of the peaks was done with reference to published literature and databases and the Chenomx spectral database contained in Chenomx NMR Suite 7.7 (Chenomx, Alberta, Canada).

### LC-MS analysis of aqueous metabolites

Half of the extracted aqueous samples were reconstituted in 0.1 ml 7:3 acetonitrile: 0.1 M aqueous ammonium carbonate containing a mixture of 8 internal standards at the concentration of 10 µM (Proline, Valine D8, Leucine D10, Lysine U13, Glutamic acid C13, Phenyl alanine D5, Succinic acid D3, Serotonin D4) (all from Sigma Aldrich except the glutamic acid from Cambridge Isotope Laboratories, MA, USA). The samples were vortexed then sonicated for 15 min followed by centrifugation at 21,000 × *g* to pellet any remaining undissolved material. They were analyzed on a Quantiva triple stage quadrupole mass spectrometer coupled to a Vanquish Horizon Ultra High Performance Chromatography (UHPLC) unit (all analytical instrument combinations supplied by Thermo Fisher Scientific), using a bridged ethylene hybrid (BEH) amide hydrophilic interaction liquid chromatography (HILIC) column^72^. The strong mobile phase (A) was 100 mM ammonium carbonate, the weak mobile phase was acetonitrile (B) with 1:1 water:acetonitrile being used for the needle wash. The LC column used was the BEH amide column (150 × 2.1 mm, 1.7 μm, Waters). The following linear gradient was used: 20% A in acetonitrile for 1.5 min followed by an increase to 60% A over 2.5 min with a further 1 min at 60% A after which the column was re-equilibrated for 1.9 min. After each chromatographic run the column was washed with 30 column volumes of 6:4 water:acetonitrile followed by a further 10 column volumes of 95:5 acetonitrile:water for storage. The total run time was 7 min, the flow rate was 0.6 mL/min and the injection volume was 5 μL. In order to resolve pentose phosphates for the identification of ribose-1-phosphate a shallower gradient was employed: 30% A in acetonitrile for 2.0 min followed by an increase to 50% A over 3.0 min with re-equilibration for 1.9 min.

The second half of the aqueous fraction was reconstituted in 0.1 mL of a 10 mM ammonium acetate water solution containing the same mixture of 8 internal standards at the concentration of 10 µM. The samples were vortexed then sonicated for 15 min followed by centrifugation at 21,000 × *g* to pellet any remaining undissolved material. They were analyzed with an ACE Excel 2 C18 PFP (100 A. 150 × 2.1 mm 5 µm) column. The electrospray voltage was set to 3500 V for the positive ionization and to 2500 V for the negative ionization. Nitrogen at 48 mTorr and 420 °C was used as a drying gas for solvent evaporation. The column was conditioned at 30 °C. The mobile phase consisted of: (A) a 0.1% of formic acid water solution and (B) a 0.1% of formic acid acetonitrile solution. The mobile phase was pumped at a flow rate of 500 µL/min programmed as follows: initially 100% of A for 1.60 min, then subjected to a linear decrease from 100% to 70% of A in 2.4 min and to 10% in 0.5 min then constant for 0.5 min and brought back to initial condition after 0.1 min.

### NMR and LC-MS data processing

NMR spectra were automatically processed in TopSpin v 3.1 (Bruker, Germany). The integrals of the different metabolites were obtained using Chenomx, normalizing to TSP and weight of the sample. LC-MS chromatograms were analyzed using Xcalibur, version 2.0 (Thermo Fisher), integrating each peak individually. while LC-MS peaks were normalized to the internal standard and weight of the sample.

### Statistics and reproducibility

For multivariate statistical analysis of metabolite profiles, datasets were imported into SIMCA-P 15.0 (Sartorius AG, Gottingen, Germany) for processing using PCA and PLS-DA (a regression extension of PCA used for supervised classification). ^1^H NMR data were Pareto scaled, in which each variable was centered and multiplied by 1/(Sk)1/2 where Sk is the standard deviation of the variable. LC–MS data were scaled to unit variance by dividing each variable by 1/(S_k_). Univariate statistical analyses were performed using Excel (Microsoft). Values are expressed as mean ± S.D. and the significance level was set at *p* < 0.05. For three groups comparison, one-way ANOVA and Tukey post-hoc test were used.*0.05 > *p* > 0.01; **0.01 > *p* > 0.001; ***p > 0.001. Sample sizes were determined on the basis of published experiments using similar methodologies and are stated in the figure legends. The experimental animals were randomly assigned to treatment groups. For all experiments, the stated replicates are biological replicates. For experimental carcinogenesis outcomes and metabolite analyses, samples were processed in random order and analyzed blinded to genotype, treatment and tissue type.

### Reporting summary

Further information on research design is available in the Nature Research Reporting Summary linked to this article.

## Supplementary information

Supplementary Information

Reporting Summary

## Data Availability

The metabolomics data have been deposited in MetaboLights, and are associated with the identifier MTBLS2961. All other data are available from the corresponding author upon reasonable request.
