# Supplementary material for: Nrf2 activation does not affect adenoma development in a mouse model of colorectal cancer
Source: Commun Biol. 2021 Sep 15;4:1081. doi: 10.1038/s42003-021-02552-w (PMC8443638; doi:10.1038/s42003-021-02552-w)
Supplement: Supplementary file 1 — Supplementary Information [file 42003_2021_2552_MOESM1_ESM.pdf]

Supplementary Information

Nrf2 activation does not affect adenoma development in a mouse model of colorectal cancer

Elena V. Knatko[a], Cecilia Castro[b], Maureen Higgins[a], Ying Zhang[a], Tadashi Honda[c], Colin J. Henderson[d], C. Roland Wolf[d], Julian L. Griffin[b,e], and Albena T. Dinkova-Kostova[a,f]

[a]Jacqui Wood Cancer Centre, Division of Cellular Medicine, School of Medicine, University of Dundee, Dundee, DD1 9SY, Scotland, UK

[b]Department of Biochemistry and the Cambridge Systems Biology Centre, University of Cambridge, Cambridge, CB2 1QW, UK

[c]Department of Chemistry and Institute of Chemical Biology & Drug Discovery, Stony Brook University, Stony Brook, NY 11794-3400, USA

[d]Division of Systems Medicine, School of Medicine, University of Dundee, Dundee, DD1 9SY, Scotland, UK

[e]Section of Biomolecular Medicine, Department of Metabolism, Digestion and Reproduction, Imperial College London, London SW7 2AZ, UK

[f]Department of Pharmacology and Molecular Sciences and Department of Medicine, Johns Hopkins University School of Medicine, Baltimore, MD 21205, USA

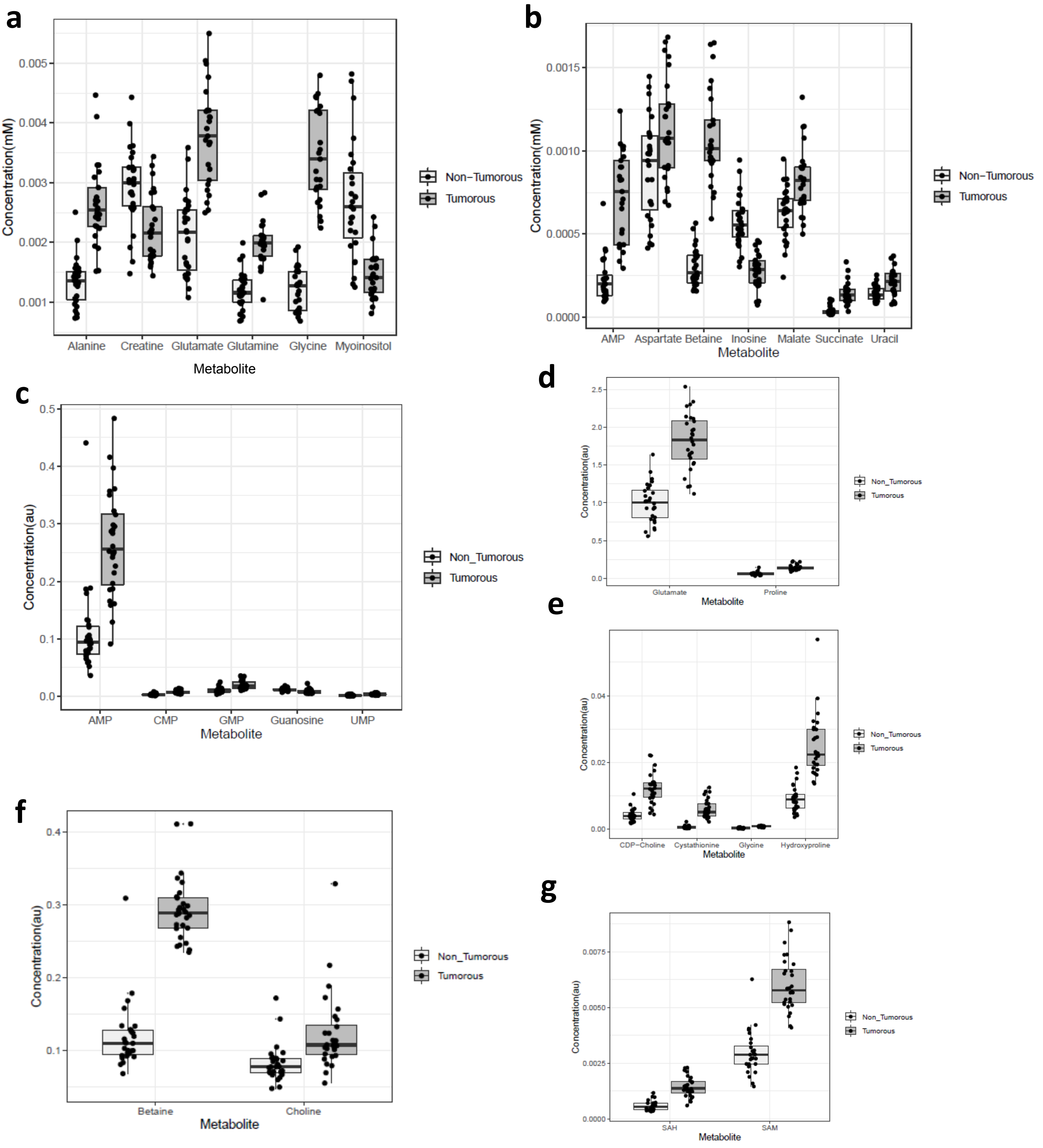

Supplementary Fig. 1. **Tumor and peri-tumoral tissues have different metabolic profiles.** (**a,b**) Concentration of selected metabolites in peri-tumorous (white bars) and tumorous (black bars) tissues as measured by NMR. In all cases p<0.001. (**c-f**) Concentration of selected metabolites in peri-tumorous (control, white bars) and tumorous (black bars) tissues as measured by LC-MS using the C18pfp-based method. For all metabolites, p<0.001.

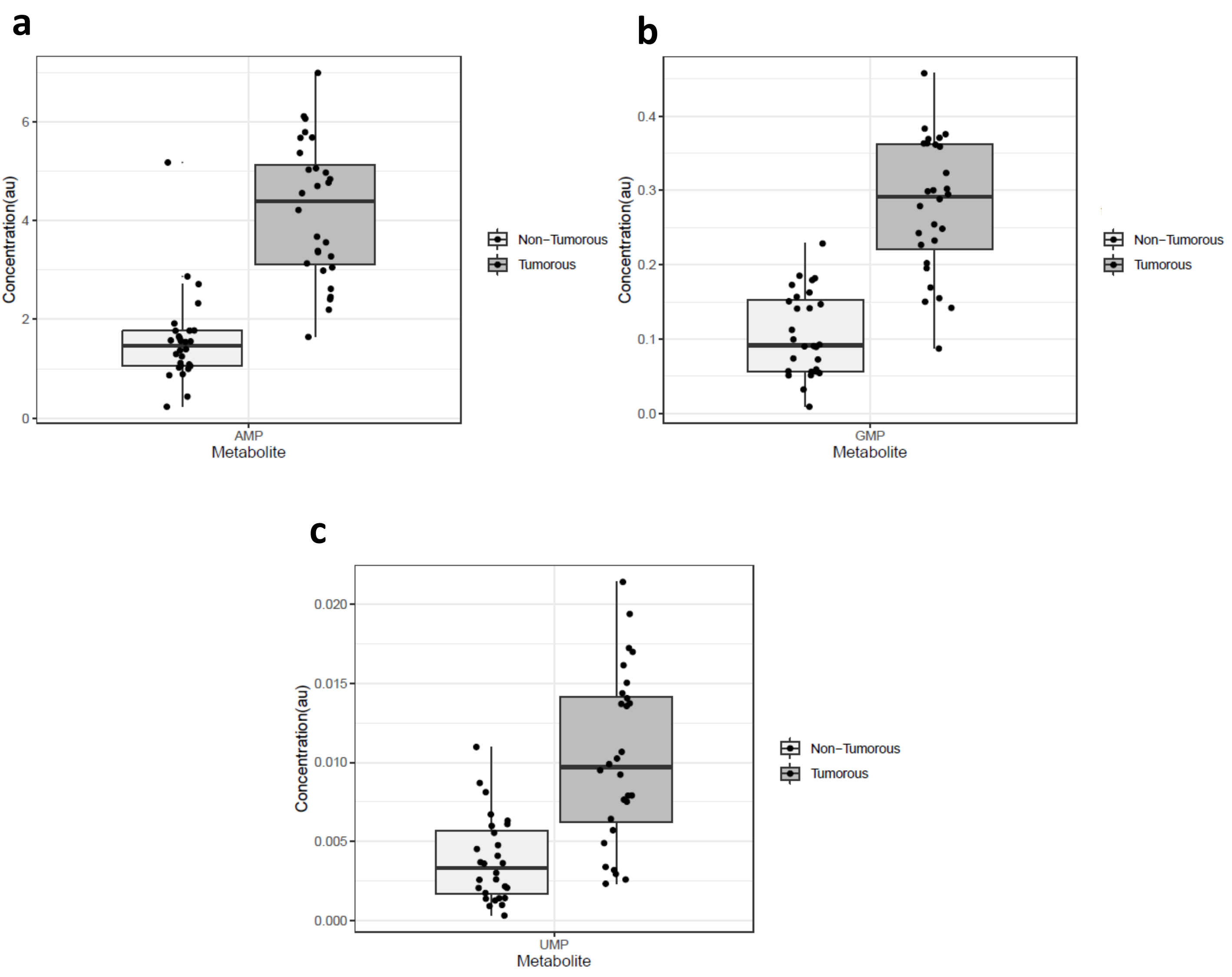

Supplementary Fig. 2. Compared to peri-tumoral tissues, tumours have increased levels of AMP, GMP and UMP. Concentration of AMP (**a**), GMP (**b**), and UMP (**c**), in tissues of peri-tumorous (white bars) and tumorous (black bars) tissues in wild-type mice as measured by LC-MS using the behamide-based method. For all metabolite, $p<0.001$.

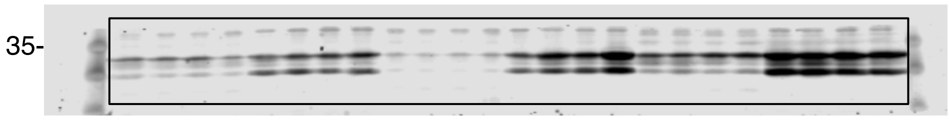

Hmox1

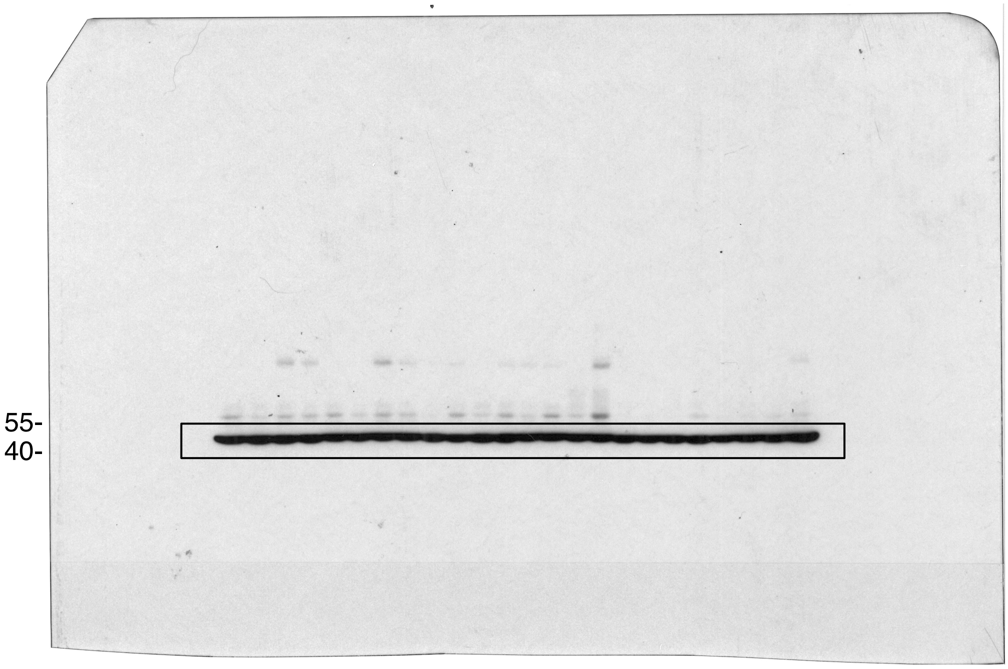

β-actin

Supplementary Fig. 3. Uncropped and unedited images of the immunoblots shown in Fig. 3i.
